# Evaluating Meniscus, Ligament and Soft Tissue Injury Using MRI in Tibial Plateau Fractures: A Tscherne Classification Approach

**DOI:** 10.3390/medicina60122073

**Published:** 2024-12-17

**Authors:** Yong-Bum Joo, Young-Mo Kim, Young-Cheol Park, Soo-Hyeok Chae, Dong-Hwan Kim

**Affiliations:** Department of Orthopedic Surgery, Chungnam National University Hospital, School of Medicine, Chungnam National University, Munhwa-ro 282, Jung-gu, Daejeon 35015, Republic of Korea; longman76@hanmail.net (Y.-B.J.); osdr69@cnu.ac.kr (Y.-M.K.); ycyeh0830@hanmail.net (Y.-C.P.); tnzur618@cnuh.co.kr (S.-H.C.)

**Keywords:** tibial plateau, tibial plateau fracture, soft tissue injury, the Tscherne classification, the Schatzker classification, magnetic resonance imaging

## Abstract

*Background and Objectives*: This study investigated associated meniscus and ligament injuries in tibial plateau fractures using magnetic resonance imaging (MRI) and assessed soft tissue injuries in relation to the Schatzker classification and Tscherne classification. *Materials and Methods*: The data of 185 patients who sustained tibial plateau fractures from January 2010 to April 2021 were retrospectively reviewed. Fractures were classified according to the Schatzker classification system. Soft-tissue injuries were assessed using the Tscherne classification. Menisci and ligaments were evaluated using preoperative MRI. Nerve injuries, compartment syndrome and wound problems were also assessed. The incidence of soft tissue injuries, as well as the relationship between the Schatzker and Tscherne classification systems, were analyzed. *Results*: Evidence of derangement of meniscus and ligament around the knee was found in 183 (98.9%) patients. The most common injury was a medial collateral ligament injury. The incidence of lateral collateral ligament injury, nerve injury, compartment syndrome and wound problem was higher in high-energy tibial plateau fractures. A tendency was observed between the Schatzker and the Tscherne classifications (*p* value < 0.001). Higher Tscherne grade was also associated with the incidence of posterior cruciate ligament injury, nerve injury and compartment syndrome. *Conclusions*: In tibial plateau fractures, soft tissue injuries were highly prevalent. High-energy fractures tended to exhibit higher Tscherne classification grades and showed an increased incidence of meniscus and ligament injuries. The Tscherne classification appears to be a helpful system for predicting soft tissue injuries in tibial plateau fractures. And preoperative MRI can be a helpful tool.

## 1. Introduction

Tibial plateau fractures are associated with a broad spectrum of injury patterns that can lead to significant complications if not managed appropriately. These fractures range from simple, isolated injury to highly complex patterns involving extensive soft tissue damage [1,2,3]. The incidence and severity of associated soft tissue injuries vary widely, with several studies reporting differing rates. Holt et al. [4] and Kode et al. [5] reported associated soft tissue injuries in tibial plateau fractures in 47.6% and 68%, respectively. Bennett and Browner [6] found 56% of soft tissue injuries, while Colletti et al. [7] stated that meniscus and ligament injuries were present in 97%. Typically, higher energy complex tibial plateau fractures are associated with severe soft tissue injuries including nerves, arteries, ligamentous and tendinous structures [8,9,10]. Stannard et al. [11] reported a high incidence of ligament injuries in high-energy groups, such as Schatzker types IV, V and VI.

Recently, the diagnosis of meniscus and ligament injury, using preoperative magnetic resonance imaging (MRI) and its relation to a treatment plan has received considerable attention [12,13,14,15]. Delamarter et al. [16] suggested that inadequate identification of the types and extent of concomitant soft tissue injuries in the treatment of tibial plateau fractures may lead to poor treatment outcomes. They stated that utilizing MRI to accurately assess the extent of soft tissue damage and guide treatment decisions can be beneficial. Nevertheless, reliable ways or objective methods for predicting the severity of soft tissue injury remains elusive [17].

In 1982, Harald Tscherne and Hans-Jorg Oestern [18,19,20] introduced the Tscherne classification for fractures, which has since become a widely referenced system for categorizing soft tissue injuries. However, there is limited research investigating concomitant soft tissue injuries in tibial plateau fractures using the Tscherne classification system.

The objectives of this study are as follows.

(1) To analyze the associated meniscus and ligament injuries according to fracture types classified by the Schatzker classification.
(2) To examine the interconnection between the Schatzker classification [2] and the Tscherne classification [18].
(3) To assess the degree of soft tissue injury in tibial plateau fractures using the Tscherne classification.

## 2. Materials and Methods

**Figure 1:**
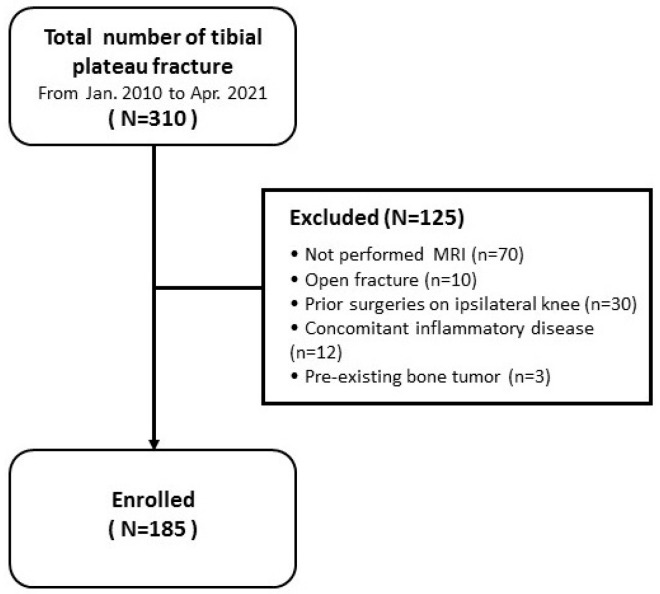
Flow diagram illustrating patient enrolment. MRI: Magnetic resonance imaging.

A total of 310 patients were diagnosed with acute tibial plateau fractures at our institution from January 2010 to April 2021. Among these, cases that did not undergo MRI before stabilizing the fracture (70 cases), open fracture (10 case), a history of trauma or surgery on the affected area (30 cases), had inflammatory diseases (12 cases) or had bone tumors (3 cases) were excluded. Thus, a total of 185 patients were evaluated retrospectively (Figure 1).

**Figure 2:**
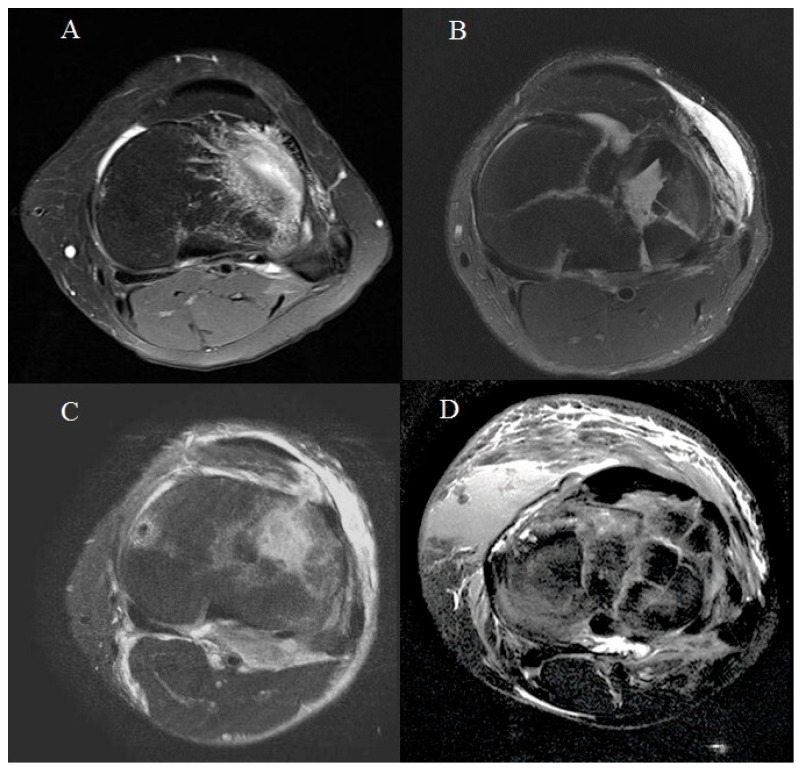
The Tscherne classification categorizes the extent of soft tissue injury associated with fracture trauma. (**A**) grade 0; none to minimal soft tissue damage, (**B**) grade 1; superficial abrasion or skin contusion, (**C**) grade 2; deep abrasion or muscle contusion, (**D**) grade 3; extensive skin contusion, crush injury with severe damage to underlying muscle, Morel-Lavallee lesion and/or vascular injury.

The fractures were classified according to the Schatzker classification by using the plain radiographs and three-dimensional computed tomography (3D CT). Knee MRI was performed before stabilizing the fracture. Preoperative MRI was performed independent of fracture type. The degree of soft tissue injury was graded according to the Tscherne classification on T2 weighted coronal MRI. In the Tscherne classification, grade 0 is none to minimal soft tissue damage; grade 1 is superficial abrasion or skin contusion; grade 2 is deep (contaminated) abrasion or muscle contusion; grade 3 is extensive skin contusion, crush injury with severe damage to underlying muscle, Morel-Lavallee lesion and/or vascular injury (Figure 2).

The MRI protocol included axial, coronal and sagittal scans with both T1- and T2-weighted sequencing. On MRI, the injuries of the menisci; the medial meniscus (MM), the lateral meniscus (LM), as well as the four ligaments, including the anterior cruciate ligament (ACL), the posterior cruciate ligament (PCL), the medial collateral ligament (MCL) and the lateral collateral ligament (LCL) were evaluated. The tibial plateau fractures were classified into two groups depending on the Schatzker classification (Group 1; low energy fracture, Schatzker type I, II, III, group 2; high-energy fracture, Schatzker type IV, V, VI). Soft tissue injury was evaluated by the Tscherne classification between the two groups. Also, the incidence of meniscus and ligament injuries were analyzed. All radiologic analyses were performed by two of the authors, both experienced trauma orthopedists with 20 and 13 years of experience, respectively, on a picture archiving and communication system workstation (Maroview, version 5.4.10.52; Marotech, Seoul, Republic of Korea). The measurements were performed two times at 4-week intervals without any patient information. Complications including nerve injuries, compartment syndrome and wound problems were also assessed. Nerve injuries were diagnosed using electromyography and nerve conduction velocity tests at least 6 weeks after trauma. Wound complications were defined as cases requiring wound revision surgery or flap surgery due to moderate to severe wound dehiscence or necrosis. Statistical analysis was conducted using SPSS version 21.0 (IBM Corp., Armonk, NY, USA). The proportion of each soft-tissue injury was calculated in relation to the total number of fractures (Table 1). Furthermore, the injury rates for each soft tissue structure were analyzed and compared with the Schatzker classification fracture group (Table 2) and Tscherne classification Grade (Table 3) with the use of the chi-square test to analyze any significant difference. The distribution of the Tscherne grades based on the Schatzker classification fracture group was compared with the use of the Fisher’s exact test in (Table 4). The incidence of nerve injury, compartment syndrome and wound problem by fracture group and the Tscherne grade was analyzed with the use of the Fisher’s exact test (Table 5). The statistical significance level was set to *p* < 0.05.

## 3. Results

The study group comprised of 117 male and 68 female patients. Patients ranged in age from 15 to 95 years (mean 54 years). Of the 185 patients, the distribution of fracture types by the Schatzker classification and the incidence of meniscus and ligament injuries of all patients are shown in Table 1. One hundred and eighty-three patients (98.9%) showed evidence of associated meniscus or ligament injury around the knee on MRI. The most common injured structure was the MCL, which was noted in 63 (34.1%) of 185 tibial plateau fractures. The breakdown of other meniscus and ligament for the whole group was as follows: MM tear; 26 (14.1%), LM tear; 40 (21.6%), ACL injury; 61 (33.0%), PCL injury; 29 (15.7%), MCL injury; 63 (34.1%), LCL injury; 28 (15.1%).

**Figure 3:**
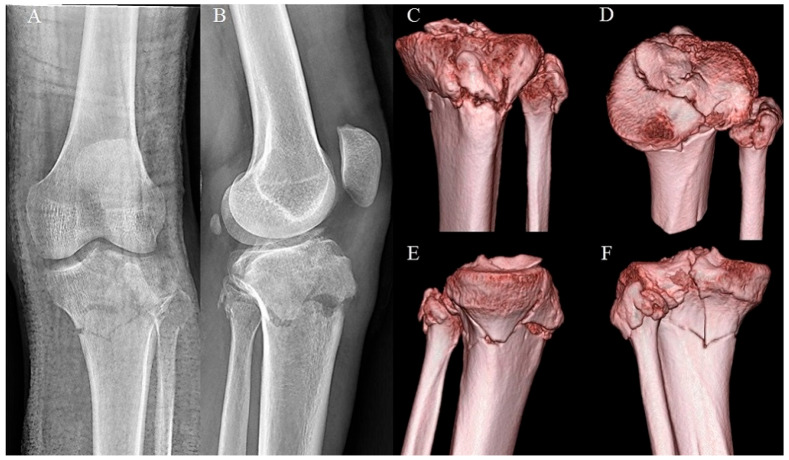
A 35-year-old male. (**A**,**B**) Preoperative anteroposterior and lateral plain X-ray shows a left proximal tibial fracture. (**C**–**F**) 3D CT shows bicondylar fracture including metaphyseal-diaphyseal discontinuity. This type is Schatzker type VI and high-energy fracture.

All patients were categorized into two groups based on the Schatzker classification: group 1 (n = 97, low-energy fractures with Schatzker type I, II, III) and group 2 (n = 88, high-energy fractures with Schatzker type IV, V, VI) (Figure 3).

The incidence of injury for each meniscus and ligament based on the group (high vs. low-energy fracture) is reported in Table 2. The incidence of LCL injury was higher in group 2 compared to group 1 (*p* = 0.006).

The incidence of the injuries of meniscus and ligament by Tscherne grade is listed in Table 3. It showed statistical difference in PCL injury (*p* = 0.036).

Table 4 presents the distribution of Tscherne grades for soft-tissue injuries according to the Schatzker classification. The severity of soft tissue injury according to the Tscherne classification was higher in group 2 (Schatzker types IV, V, VI) compared to group 1 (Schatzker type I, II, III) (*p* < 0.001).

The incidence of nerve injury, compartment syndrome and wound problem for each fracture group and Tscherne grade were listed in Table 5. The incidence of nerve injury, compartment syndrome, and wound problems was higher in group 2 than in group 1. (all *p* < 0.05) Also, the incidence of nerve injury and compartment syndrome was higher in Tscherne grade 3 than grade 1 and 2 (all *p* < 0.05).

## 4. Discussion

Meniscus and ligament injuries are prevalent among patients with tibial plateau fractures. Delamarter et al. [16] found that 22% of patients with tibial plateau fractures have collateral or cruciate ligaments damage coexisting. Blokker et al. [21] reported 20 patients (31%) with meniscus and ligament injuries in 64 patients with tibial plateau fractures. Among 94 patients, Schatzker et al. [2] reported only eight ligament injuries. Gardner et al. [22] reported a 99% incidence of concomitant soft tissue injury and reported 77% of complete ligament rupture in 103 patients with proximal tibia fractures. In this study, 183 patients (98.9%) showed evidence of derangement of meniscus and ligament. These results suggest that the frequency of soft tissue injuries associated with tibial plateau fractures may be higher than previously reported.

The most common injured structures associated with tibial plateau fractures was MCL injuries, noted in 63 (34.1%) of 185 cases (Table 1). Several previous studies have reported similar findings, indicating that MCL injuries are the most prevalent injured structure in tibial plateau fractures. Bennett and Browne [6] reported MCL injuries in 6 out of 30 cases (20%), Colletti et al. [7] in 16 out of 29 cases (55%), and Delamarter et al. [16] in 24 out of 39 cases (61.5%). Each study identified MCL injuries as the most frequent. The relatively high incidence of MCL injuries in this study may be attributed to the high frequency of lateral tibial plateau fractures resulting from valgus forces. It is well known that both axial and valgus forces causing lateral plateau fractures may lead to MCL injuries [23]. In this study, lateral tibial plateau fractures (Schatzker types I, II, and III) accounted for 97 of 185 cases (52.4%), which was higher than the incidence of medial or bicondylar fractures. Moreover, MCL injuries are known to commonly occur in Schatzker type II fractures. The most common fracture type in this study was Schatzker type II, representing 53 of 185 cases (28.6%), which may explain the relatively high frequency of MCL injuries observed in our study.

In this research, the high-energy fracture group in the Schatzker classification, compared to the low-energy fracture group, exhibited a higher frequency of LCL injury (Table 2). This finding aligns with previous research results. Stannard et al. [11] reported a high frequency of soft tissue injuries including menisci, ACL, PCL, the posteromedial corner (PMC), and the posterolateral corner (PLC) in tibial plateau fractures. They noted that 78% of the patients with high-energy patterns (Schatzker IV, V, VI) had torn ligaments compared with only 46% of patients with low-energy patterns (Schatzker I, II, III) indicating an increasing incidence of ligament injury with the severity of bony damage. Furthermore, Thürig et al. [12] demonstrated through a meta-analysis that the frequency of LCL and ACL injuries significantly increases with the severity of the Schatzker classification fracture types.

The results demonstrated a tendency for high-energy fractures to exhibit a higher Tscherne classification and higher incidence of meniscus and ligament injuries. More than half of the patients with high-energy tibial plateau fractures exhibited Tscherne grade 3 (65.9%) (Table 4). This indicates that, in the Schatzker classification, the severity of soft tissue damage to skin, muscles, increases with the energy level of the injury.

In the analysis of the incidence of meniscus and ligament injuries according to Tscherne grade, although there was no statistical significance, there was a tendency for the frequencies of MM, LM, ACL and LCL injuries to increase with higher Tscherne grades (Table 3). A higher Tscherne grade indicates a high-energy injury mechanism and more complex fracture patterns, which may result in greater intra-articular structure damage. It is widely recognized that high-energy injuries can result in comminuted fractures accompanied by extensive damage to bone, soft tissues and neurovascular structures [24]. Stannard et al. [11] showed in their paper that the incidence of ligament injury increases with the severity of bony injury. The Tscherne classification system is based on the physiological principle that the energy exerted to the bone, which determines the fracture pattern, is directly related to the energy transferred to the adjacent soft tissues [18,19]. Thus, the statistical relationship between the Tscherne and Schatzker classifications in this result is due to higher energy fracture patterns, such as those seen in Schatzker types IV, V, and VI, being associated with more severe soft tissue injuries. This increase in injury severity is reflected in the Tscherne classification system, where higher energy corresponds to higher Tscherne grades.

**Figure 4:**
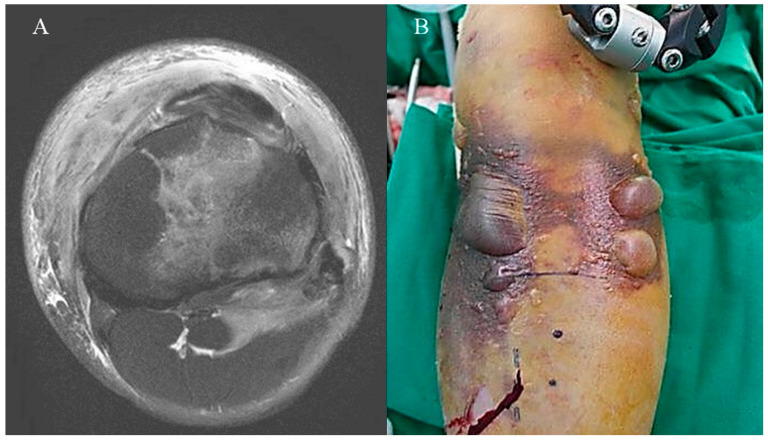
Same patient with Figure 2 (**A**) Preoperative MRI shows soft tissue injury of the Tscherne classification grade 3. (**B**) On day 1 after injury, the patient’s lower leg was severely swollen and showed hemorrhagic fracture blisters.

**Figure 5:**
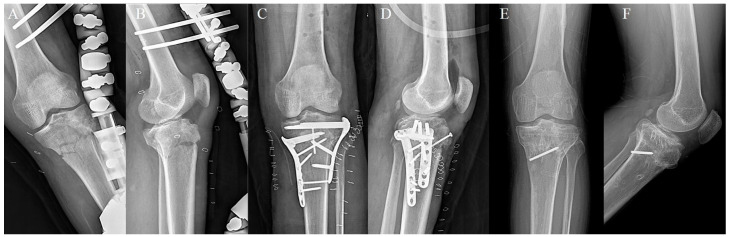
Same patient with Figure 2 (**A**,**B**) Temporary external fixation was done. (**C**,**D**) 20 days after external fixator application, dual locking compression plate fixation was done. (**E**,**F**) 18 months after plate application, bony union was seen and all metal materials except one broken screw were removed.

Based on the results, there was a tendency between the incidence of PCL injuries and the Tscherne grade (Table 3). Unlike ACL injuries, which are primarily associated with athletic activities, the majority of PCL injuries result from high-energy trauma [25]. More violent and high-speed mechanisms, such as vehicle and motorcycle accidents or falls from heights injury, are more likely to result in PCL injuries [26]. The findings indicate that a higher Tscherne grade correlates with an increased incidence of PCL injuries. Thus, it may be inferred that high-energy trauma produces a greater frequency of PCL injuries with higher Tscherne grade. Furthermore, it was found that as Tscherne grade increased, the frequency of nerve injury and wound complications significantly increased (Table 5). This finding is consistent with previous studies that report an increase in the frequency and severity of vascular and nerve injuries, as well as compartment syndrome, with higher-energy injuries [27]. And furthermore, Ibrahim et al. [19] also noted that primary definitive fixation is appropriate for lower-grade soft tissue injuries, such as Tscherne grade 0 or 1, whereas staged treatment is more appropriate for higher-grade soft tissue injuries, such as Tscherne grade 2 or 3 (Figure 4 and Figure 5). Thus, the Tscherne classification appears to be a potentially helpful method for evaluating associated soft tissue injuries in tibial plateau fractures. It may serve as a reference in determining treatment strategies.

The results of this study suggest the frequency of soft tissue injuries associated with tibial plateau fractures is significantly high. Virtually all cases (98.9%) were found to involve soft tissue injuries. Consequently, it is essential to suspect some degree of soft tissue injury in all patients with tibial plateau fractures and to make diligent efforts not to overlook these injuries during the diagnostic process. Given the high frequency, a comprehensive understanding of the overall injury pattern in patients with tibial plateau fractures is crucial. This involves not only assessing the fracture itself but also meticulously considering the extent and the nature of the associated soft tissue injuries. Therefore, accurate preoperative diagnosis of soft tissue injuries can aid surgeons in devising appropriate treatment plans and selecting suitable surgical approaches. It can also significantly help in preventing postoperative complications. This study highlights the importance of integrating soft tissue injury assessment into fracture management protocols. Future research could explore the broader applicability of preoperative MRI and refine injury classification systems to improve patient outcomes.

This study has several limitations. First, it is a retrospective study with a small sample size conducted at a single institution, so the results may differ in larger-scale or multi-center studies. However, to the authors’ knowledge, this study has a relatively large sample size among studies analyzing soft tissue injuries in tibial plateau fractures. Second, an analysis of the patterns of ligament injuries and meniscus tears was not performed. Nevertheless, only traumatic lesions were analyzed, excluding degenerative meniscus tear findings on MRI, to focus on their association with acute fractures. Third, as a radiological study, this study did not analyze the clinical outcomes between patients with and without meniscus and ligament injuries. Fourth, the intraclass correlation coefficient between two observers were not calculated.

## 5. Conclusions

Compared to the low-energy tibial plateau fracture group in the Schatzker classification, the high-energy fracture group exhibited a higher frequency of lateral collateral ligament injury, nerve injury, compartment syndrome and postoperative wound complications. There was a tendency to observe between higher energy fracture group in the Schatzker classification and higher-grade soft tissue injury in the Tscherne classification. The Tscherne classification appears to be a helpful system for predicting soft tissue injuries in tibial plateau fractures. In addition, preoperative MRI can be a potentially helpful tool for identifying associated soft tissue injuries and in planning the treatment approach, which is crucial for deciding the treatment plan in the management of tibial plateau fractures. As a result, surgeons should consider utilizing MR imaging for high energy tibial plateau fractures to characterize the overall injury.

## Figures and Tables

**Table 1:** The Overall Incidence of Meniscus and Ligament Injury Based on the Schatzker Classification.

Schatzker Type	Number of Patients, n(%)	Menisci, n (%)		Cruciate Ligament, n (%)		Collateral Ligament, n (%)	
		MM	LM	ACL	PCL	MCL	LCL
1	15 (8.1%)	4 (26.7%)	4 (26.7%)	4 (26.7%)	4 (26.7%)	5 (33.3%)	0 (0.0%)
2	53 (28.6%)	4 (7.5%)	14 (26.4%)	16 (30.2%)	4 (7.5%)	22 (41.5%)	5 (9.4%)
3	29 (15.7%)	4 (13.8%)	4 (13.8%)	10 (34.5%)	5 (17.2%)	11 (37.9%)	3 (10.3%)
4	23 (12.4%)	3 (13.0%)	1 (4.3%)	6 (26.1%)	6 (26.1%)	7 (30.4%)	8 (34.8%)
5	43 (23.2%)	9 (20.9%)	12 (27.9%)	20 (46.5%)	6 (1.0%)	11 (25.6%)	10 (23.3%)
6	22 (11.9%)	2 (9.1%)	5 (22.7%)	5 (22.7%)	4 (18.2%)	7 (31.8%)	2 (9.1%)
Total n (%)	185 (100%)	26 (14.1%)	40 (21.6%)	61 (33.0%)	29 (15.7%)	63 (34.1%)	28 (15.1%)

Abbreviations: MM, Medial meniscus; LM, Lateral meniscus; ACL, anterior cruciate ligament; PCL, posterior cruciate ligament; MCL, medial collateral ligament; LCL, lateral collateral ligament.

**Table 2:** The Overall Incidence of Meniscus and Ligament Injury Based on Low or High Energy Fracture Group.

Schatzker Type	Menisci, n (%)		Cruciate Ligament, n (%)		Collateral Ligament, n (%)	
	MM	LM	ACL	PCL	MCL	LCL
* Group 1 (n = 97)	12 (9.4%)	22 (22.7%)	30 (30.9%)	13 (13.4%)	38 (39.2%)	8 (8.2%)
* Group 2 (n = 88)	14 (13.0%)	18 (20.5%)	31 (35.2%)	16 (18.2%)	25 (28.4%)	20 (22.7%)
Total, n (%)	26 (14.1%)	40 (21.6%)	61 (33.0%)	29 (15.7%)	63 (34.1%)	28 (15.1%)
*p* value	0.489	0.713	0.534	0.372	0.123	0.006

Abbreviations: MM, Medial meniscus; LM, Lateral meniscus; ACL, anterior cruciate ligament; PCL, posterior cruciate ligament; MCL, medial collateral ligament; LCL, lateral collateral ligament; * Group 1 = Schatzker type I, II, III; * Group 2 = Schatzker type IV, V, VI.

**Table 3:** The Incidence of Meniscus and Ligament Injury by Tscherne Grade.

Tscherne Grade	Menisci, n (%)		Cruciate Ligament, n (%)		Collateral Ligament, n (%)	
	MM	LM	ACL	PCL	MCL	LCL
Grade 1	3 (9.4%)	6 (18.8%)	10 (31.3%)	1 (3.1%)	8 (25.0%)	1 (3.1%)
Grade 2	12 (13.0%)	18 (19.6%)	29 (31.5%)	20 (21.7%)	39 (42.4%)	14 (15.2%)
Grade 3	11 (18.0%)	16 (26.2%)	22 (36.1%)	8 (13.1%)	16 (26.2%)	13 (21.3%)
Total, n (%)	26 (14.1%)	40 (21.6%)	61 (33.0%)	29 (15.7%)	63 (34.1%)	28 (15.1%)
*p* value	0.483	0.563	0.821	0.036	0.058	0.067

Abbreviations: MM, Medial meniscus; LM, Lateral meniscus; ACL, anterior cruciate ligament; PCL, posterior cruciate ligament; MCL, medial collateral ligament; LCL, lateral collateral ligament.

**Table 4:** The Distribution of Tscherne Grades Based on High and Low Energy Fracture Group.

Schatzker Type	Tscherne Grade 1	Tscherne Grade 2	Tscherne Grade 3	Total (n)	*p* Value
* Group 1	30 (30.9%)	64 (66.0%)	3 (3.1%)	97 (52.4%)	<0.001
* Group 2	2 (2.3%)	28 (31.8%)	58 (65.9%)	88 (47.6%)	
Total, n (%)	32 (17.3%)	92 (49.7%)	61 (33.0%)	185 (100%)	

* Group 1 = Schatzker type I, II, III; * Group 2 = Schatzker type IV, V, VI.

**Table 5:** The Incidence of Nerve Injury, Compartment Syndrome and Wound Problem by High and Low Energy Fracture Group and Tscherne Grade.

	^†^ Nerve Injury, n (%)	Compartment Syndrome, n (%)	Wound Problem, n (%)
Fracture group by Schatzker type			
* Group 1	1 (1.0%)	0 (0%)	2 (2.0%)
* Group 2	7 (8.0%)	4 (4.6%)	9 (10.3%)
*p* value	0.028	0.049	0.027
Tscherne grade			
Grade 1	0 (0%)	0 (0%)	0 (0%)
Grade 2	2 (2.2%)	0 (0%)	5 (5.4%)
Grade 3	6 (9.8%)	4 (6.6%)	6 (9.8%)
*p* value	0.033	0.028	0.161

* Group 1 = Schatzker type I, II, III; * Group 2 = Schatzker type IV, V, VI. ^†^ Nerve injury: of the total 8 cases, 7 cases were common peroneal nerve and 1 case was concomitant common peroneal and tibial nerve injury.

## Data Availability

Dataset available on request from the authors.
